# Decavanadate-Bearing Guanidine Derivatives Developed as Antimicrobial and Antitumor Species

**DOI:** 10.3390/ijms242417137

**Published:** 2023-12-05

**Authors:** Andreea Dumitrescu, Catalin Maxim, Mihaela Badea, Arpad Mihai Rostas, Alexandra Ciorîță, Alina Tirsoaga, Rodica Olar

**Affiliations:** 1 Department of Inorganic and Organic Chemistry, Biochemistry and Catalysis, Faculty of Chemistry, University of Bucharest, 90-92 Panduri Str., District 5, 050663 Bucharest, Romania; andreea.dumitrescu@s.unibuc.ro (A.D.); catalin.maxim@chimie.unibuc.ro (C.M.); mihaela.badea@chimie.unibuc.ro (M.B.); 2 Department of Physics of Nanostructured Systems, National Institute for Research and Development of Isotopic and Molecular Technologies, 67-103 Donat Str., 400293 Cluj-Napoca, Romania; arpad.rostas@itim-cj.ro; 3 Department of Molecular Biology and Biotechnology, Faculty of Biology and Geology, Babeș-Bolyai University, 5-7 Clinicilor St., 400001 Cluj-Napoca, Romania; 4 Department of Analytical and Physical Chemistry, Faculty of Chemistry, University of Bucharest, 4-12 Regina Elisabeta Av., District 3, 030018 Bucharest, Romania; alina.jurca@unibuc.ro

**Keywords:** antimicrobial, cytotoxicity, decavanadate, DFT calculations, guanidine, guanylurea

## Abstract

To obtain biologically active species, a series of decavanadates (Hpbg)_4_[H_2_V_10_O_28_]·6H_2_O (**1**) (Htbg)_4_[H_2_V_10_O_28_]·6H_2_O; (**2**) (Hgnd)_2_(Hgnu)_4_[V_10_O_28_]; (**3**) (Hgnu)_6_[V_10_O_28_]·2H_2_O; and (**4**) (pbg = 1-phenyl biguanide, tbg = 1-(*o*-tolyl)biguanide, gnd = guanidine, and gnu = guanylurea) were synthesized and characterized by several spectroscopic techniques (IR, UV-Vis, and EPR) as well as by single crystal X-ray diffraction. Compound (**1**) crystallizes in space group *P*-1 while (**3**) and (**4**) adopt the same centrosymmetric space group *P*21/n. The unusual signal identified by EPR spectroscopy was assigned to a charge-transfer π(O)→d(V) process. Both stability in solution and reactivity towards reactive oxygen species (O_2_^−^ and OH·) were screened through EPR signal modification. All compounds inhibited the development of *Escherichia coli*, *Pseudomonas aeruginosa*, *Staphylococcus aureus*, and *Enterococcus faecalis* bacterial strains in a planktonic state at a micromolar level, the most active being compound (**3**). However, the experiments conducted at a minimal inhibitory concentration (MIC) indicated that the compounds do not disrupt the biofilm produced by these bacterial strains. The cytotoxicity assayed against A375 human melanoma cells and BJ human fibroblasts by testing the viability, lactate dehydrogenase, and nitric oxide levels indicated compound (**1**) as the most active in tumor cells.

## 1. Introduction

The disease evolution in the modern epoch requests the diversification of treatment approaches. For instance, bacterial infections are increasingly resistant to treatment with antibiotics [1,2,3] or hard to treat due to complications with biofilms, both mono- and poly-microbial ones [4,5,6]. On the other hand, both organic and inorganic cytostatics used in the current treatment of cancer are expensive and generate resistance. Moreover, these have limited effectiveness, especially for recurrent and metastatic cancers, and are highly toxic due to their unspecific mechanisms of action [7,8,9]. As a result, new approaches are required for these diseases’ treatment, one involving inorganic–organic hybrid species such as polyoxometalates (POMs) [10,11,12].

Among POMs, decavanadates ([H_n_V_10_O_28_]^(n−6-)^, (n = 0–4)) (DVs) are oxo-clusters that involve ten distorted VO_6_ octahedra sharing edges and vertices [13]. These species exhibit in structure V(V) ions linked by oxo anions that act as a bridge between two, three, or four metallic ions. The structure is completed by some anions involved in double bonds as terminal ligands. Because of the high anionic charge, DV can be mainly stabilized in a solid state by counterions through electrostatic interactions. As a result, DV can be combined with various cations, including proton, representative and transition metal ions, ammonium, cationic complexes, alkylammonium, phosphonium, and organic or organometallic species [10,11,12,13].

Moreover, DV in combination with different inorganic and organic cations or a mixture of them generates species with a wide range of biological activities, such as hypoglycemic, antimicrobial, anticancer, or antiviral.

Like other V(V) derivatives, DVs were first studied as insulin-mimetic species [10]. Hence, ammonium DV (ADV) exhibits insulin-like activity and enhances glucose uptake in the presence of insulin [14]. Moreover, the activity was improved for benzyl ammonium [15] or *N*,*N*’-dimethylbiguanidinium DVs [16].

On the other hand, the DVs combined with ammonium [17], ammonium and [M(OH_2_)_6_]^2+^ (M = Ni [18,19], Co [19], Mg [20]) or hexamethylenetetramine [21], and 3/4-pyridinium carboxamide [22] cations inhibit the growth of some Gram-negative bacteria like *Escherichia coli* [17,18,19,20,21,22] or *Pseudomonas aeruginosa* [19,20] in a micromolar range. For other species with protonated 4-picolinic acid [23], sodium [24], or calcium [25] cations, the inhibition spectrum was extended against Gram-positive bacteria, such as *Bacillus ciroflagellosus* [23], *Mycobacterium smegmatis* [24], *Mycobacterium tuberculosis* [24], and *Staphylococcus aureus* strains [25]. These studies evidenced that DVs are generally more effective than mono-, di- and tetra vanadate, which rapidly decompose in biological media [10,11]. The enhanced activity can be related to many V(V) centers and general properties such as total net charge, size, and redox activity.

Concerning the antitumor activity, the DVs bearing Co(II) and sodium cations inhibit cell proliferation in both human liver (SMMC-7721) and ovary (SKOV-3) cancer cell lines, a better activity compared to the approved drug fluorouracil [26]. At the same time, species with ammonium and lithium demonstrated dose-dependent antiproliferative activity on glioblastoma (U87), breast (MDA-MB-231), and melanoma (IGR39) human invasive cell lines [27]. The same cancer cell lines were inhibited in a dose-dependent manner by DV with mixed [Mg(H_2_O)_6_]^2+^ and 2-methylimidazolium species [28].

Using the organic cations *N*,*N*,*N*′,*N*′-tetramethylethylenediamine, and ethylenediamine, some DVs exhibiting very good activity against human lung carcinoma (A549) and murine leukemia (P388) cells were obtained. It is worth mentioning that against A549 cells, both DVs exhibit inhibition values lower in comparison with cisplatin, but they have cytotoxicity on normal human hepatocytes (LO2) [29]. Unlike these, species with both sodium and carnitine do not affect normal cells, but the cytotoxicity against A549 is lower compared to the cisplatin one. In addition, this compound is 400 times more active against the MDA-MB-231 cell line than cisplatin [30]. The exposure of human breast epithelial (MCF-7) and A549 cancer cells to trimethyl ammonium acetate DV reduced the cell viability in a concentration-dependent fashion [31], while ADV and *N*,*N′*-dimethylbiguanidine DV exert an antiproliferative effect on the human melanoma cell line (UACC-62) at lower concentrations than ammonium metavanadate [32]. Moreover, the tetra-(benzyl ammonium) dihydrogen DV inhibited the proliferation and migration of MDA-MB231 at a micromolar range [33].

Moreover, the ability of ADV to associate with the receptor-binding domain of the SARS-CoV-2 spike protein and disrupt the protein’s binding to its host cell surface receptor was recently demonstrated. Unfortunately, in vitro studies on SARS-CoV-2 infected cells identify enhanced ADV cytotoxicity [34].

All these results demonstrated both the potential of DVs for medical applications and the fact that the biological activity can be fine-tuned through single- or mixed-type counter-cations.

Considering the known biological activity of guanidine derivatives [35] and the proven antitumor activity of *N*,*N*’-dimethylbiguanidinium DV [32], the biological potential of some new DV-bearing guanidine cations was studied to obtain species with good antimicrobial or antitumor activities. Moreover, the anti-biofilm potential of such compounds is first reported here. DVs bearing guanidium derivatives were fully characterized based on data provided by single X-ray diffraction.

## 2. Results and Discussion

**Scheme 1:**
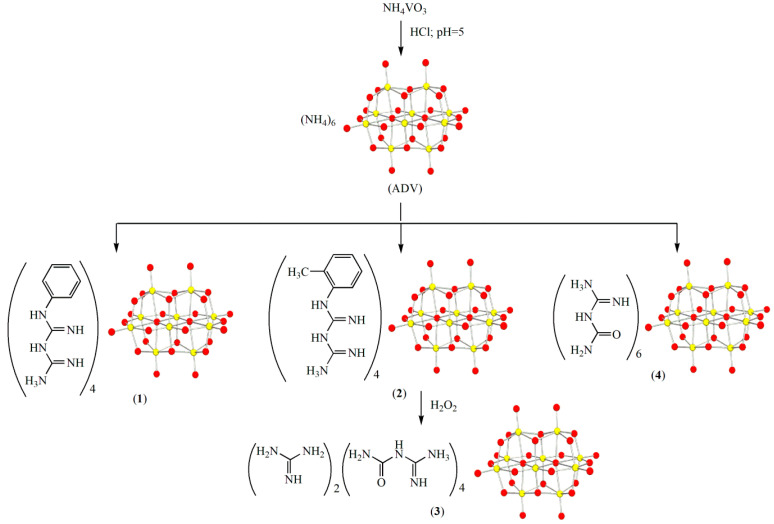
Synthesis route of the decavanadates: ADV, (Hpbg)_4_[H_2_V_10_O_28_]·6H_2_O (**1**), (Htbg)_4_[H_2_V_10_O_28_]·6H_2_O (**2**), (Hgnd)_2_(Hgnu)_4_[V_10_O_28_] (**3**), and (Hgnu)_6_[V_10_O_28_]·2H_2_O (**4**) (in DV structure the red dots represent vanadium atoms and yellow dots oxygen atoms).

The DVs were prepared in a two-step procedure. First, the ADV was prepared by adding HCl in an aqueous solution of ammonium metavanadate up to pH 5, and a biguanide in a proper ratio was added (Scheme 1). For 1-phenyl biguanide (pbg) and 1-(*o*-tolyl)biguanide (tbg), the isolated species correspond to (Hpbg)_4_[H_2_V_10_O_28_]·6H_2_O (**1**) and (Htbg)_4_[H_2_V_10_O_28_]·6H_2_O (**2**). By adding hydrogen peroxide to the system containing tbg, an interesting compound bearing both cations of guanidine (gnd) and guanylurea (gnu), (Hgnd)_2_(Hgnu)_4_[V_10_O_28_] (**3**) was isolated. These cations were generated by the oxidative cleavage of tbg and water addition to one of the resulting species. In an attempt to obtain the DV with 1-cyanoguanidine, the compound (Hgnu)_6_[V_10_O_28_]·2H_2_O (**4**) was isolated, containing the same cation as guanylurea, this time by water addition to the guanidine derivative.

The compounds were characterized through a plethora of physicochemical methods, both in solid state and in solution. Compounds (**1**), (**3**), and (**4**) were fully characterized through single crystal X-ray diffraction. Moreover, all species were screened comparatively with ADV for biological activity. All these findings are detailed below.

### 2.1. Description of the Crystal Structure of Compounds

For compounds, (**1**), (**3**), and (**4**) single crystals suitable for X-ray analysis were obtained. This method provided information regarding the molecular structure and non-covalent interactions that appear between component moieties.

The crystallographic data and experimental details associated with the newly synthesized compounds are detailed in Supplementary Table S1. The selected bond distances and angles for all species are shown in Supplementary Tables S2–S7.

**Figure 1:**
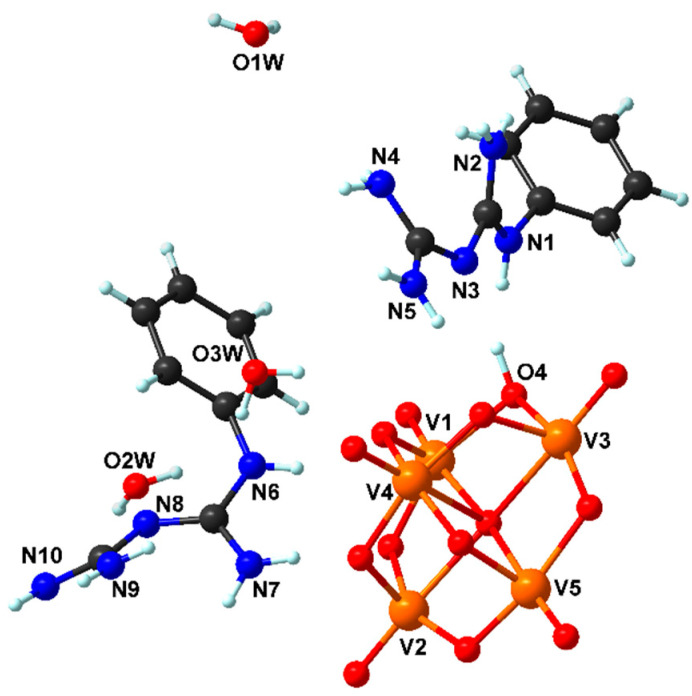
The asymmetric unit with the atom-labelling scheme of (**1**).

For (**1**) the (H_2_V_10_O_28_)^4−^ anion is situated on an inversion center in space group *P*-1 and approaches the expected D_2h_ symmetry, which has already been reported. The asymmetric unit for (**1**), presented in Figure 1, also contains two crystallographically independent phenyl biguanide cations and one water molecule.

The Hpbg^+^ monocation has its charge located mainly on the triangles N3N4N5 and N8N9N10. Furthermore, this cation is characterized by a dihedral angle of 73.02° and 22.5° between these planes and the phenyl ring.

**Figure 2:**
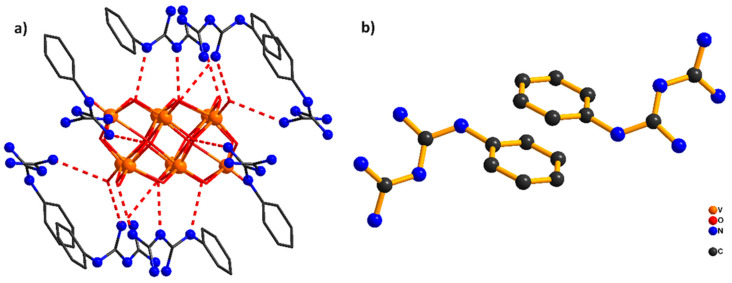
Perspective view of the crystal structure of (**1**) showing (**a**) hydrogen bond interactions between anionic and cationic units and (**b**) noncovalent π–π interactions.

The decavanadate anion, the cations, and the crystal water molecules engage in an extensive network of hydrogen bonds (Figure 2a). All N–H and O–H groups present in the asymmetric unit serve as donor groups. The two strongest hydrogen bonds are formed between the anion V_10_-μ_3_OH and one nitrogen from the cation (N3–O6 = 2.743). A packing diagram showing the π–π interactions between the phenyl rings of the biguanide cations is presented in Figure 2b. The distances between aromatic moieties are 3.68 Å.

**Figure 3:**
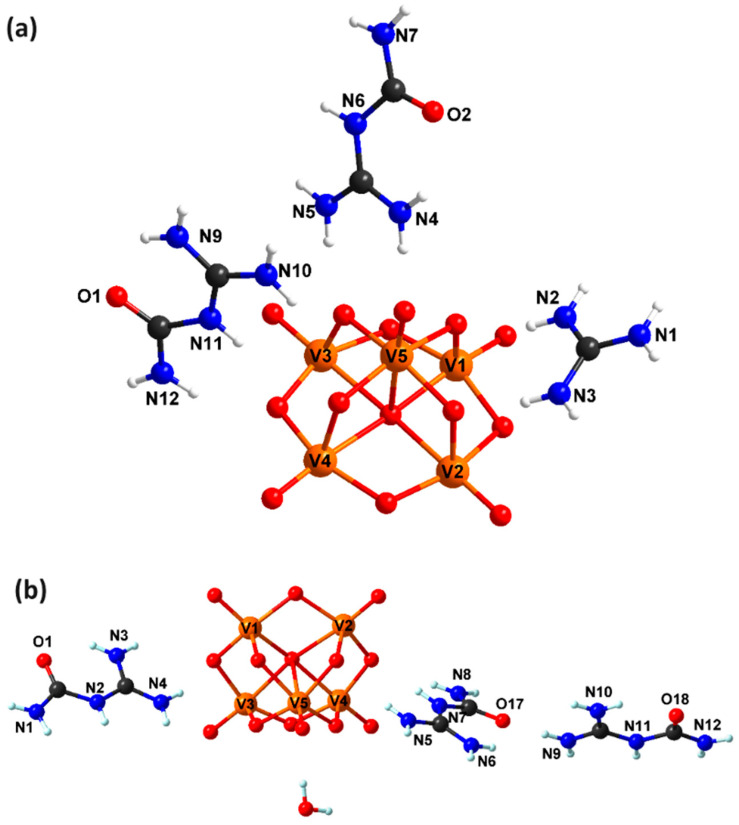
The asymmetric unit with the atom-labelling scheme of (**3**) (**a**) and (**4**) (**b**).

By replacing the pbg with 1-(*o*-tolyl)biguanide, in different reaction conditions, two compounds (Hgnd)_2_(Hgnu)_4_[V_10_O_28_] (**3**) and (Hgnu)_6_[V_10_O_28_]·2H_2_O (**4**) have been crystallized. These compounds crystallize in the same centrosymmetric space group *P*2_1_/n, with the [V_10_O_28_]^6−^ anion lying in an inversion center. The difference in the asymmetric units (Figure 3) of these compounds is the presence of different cations and solvent molecules.

For (**3**), the apparition of guanylurea and guanidinium cations was observed, while for (**4**), only guanylurea and crystallization water molecules were observed.

In the two compounds, the V_10_ is built with three types of vanadium atoms with different environments as follows: (i) two V atoms in the center (V_1_), each binding to six bridging O atoms, including both of the μ_6_-O atoms of the complex; (ii) four V atoms (V_2,3_), each corresponding to one μ_6_-O atom, four μ_2_-O atoms, and a terminal O atom; and (iii) four V atoms (V_4,5_), each with five bridging O atoms and one terminal O atom.

**Figure 4:**
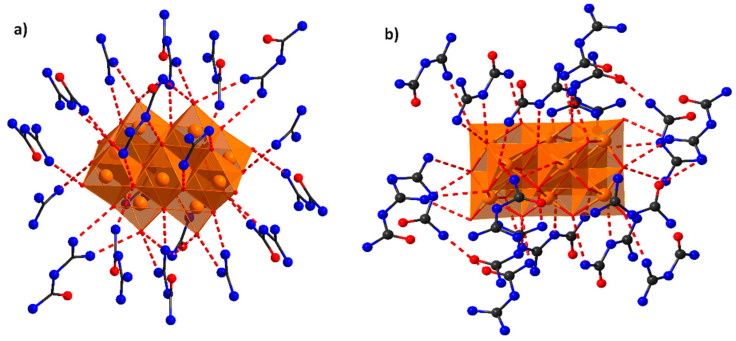
Extended hydrogen bond networks in compounds (**3**) (**a**) and (**4**) (**b**).

The resulting 3D hydrogen network (Figure 4) is complicated because of the presence of different cations and crystallizations of water molecules. Practically all the amines, guanidinium, water molecules, and oxo donor groups are engaged in hydrogen bonds. In particular, the guanidinium cations interact with decavanadate anions in (**3**). Thus, the presence of planar cations within the lattice results in several NH donors with different orientations compared to those of the donors found in the phenyl-guanidine cation, and this supplies these V(V) clusters with more unpredictability in the composition and nature of the hydrogen-bond interactions.

### 2.2. Physicochemical Characterization of Decavanadates

#### 2.2.1. FT-IR Spectra

The IR spectra of DVs were studied to obtain information concerning the characteristic bands associated with the main functional groups of guanidine derivatives and DV core, respectively.

**Figure 5:**
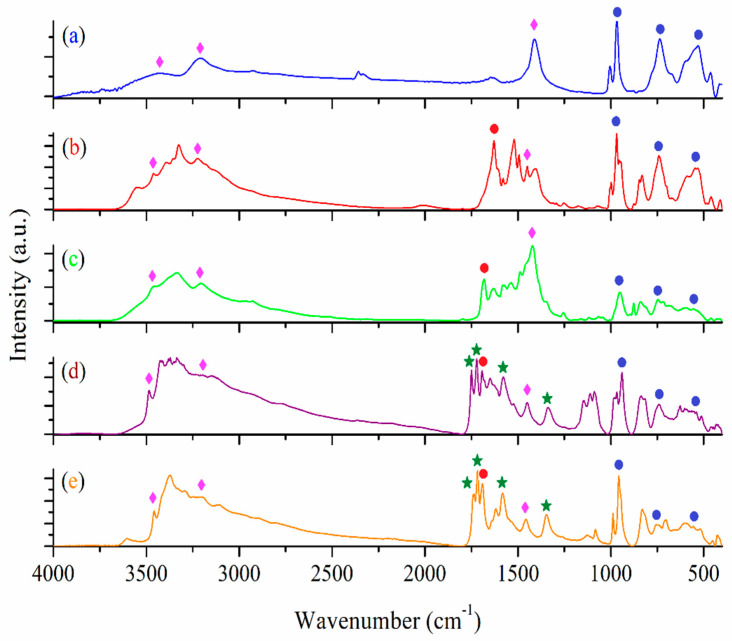
IR spectra of compounds ADV (**a**), (**1**) (**b**), (**2**) (**c**), (**3**) (**d**), and (**4**) (**e**) registered in KBr pellets. The characteristic bands of the DV core were highlighted with blue circles, the ammonium group with magenta rhomb, the C=N group of the guanidine moiety with red circles, and the amide group of the urea moiety with green stars.

These spectra for ADV and the (**1**–**4**) species are depicted in Figure 5.

All spectra show characteristic vibration bands of all building units, DV, and the corresponding cations. The strong IR band at around 950 cm^−1^ is assigned to the stretching vibration of the V=O group. The asymmetric and symmetric vibrations of the bridge V–O–V groups appear in the 730–880 cm^−1^ range, while the vibration of the V–O group is around 515 cm^−1^ [18,19,20,21,22,23,24,25,26,27,28,29,30,31,32,33]. The spectra exhibit the characteristic bands of ammonium cations in the 3200–3460 and 1410–1450 cm^−1^ ranges [21]. The band assigned to stretching vibration of the C=N bond from the guanidine moiety appears in the 1620–1690 cm^−1^ range. For complexes (**3**) and (**4**), the spectra are more complex since they contain supplementary bands, two in the 1710–1755 cm^−1^ range and one around 1580 and 1340 cm^−1^ that arise from combined vibration modes for the amide group [36].

#### 2.2.2. UV-Vis Spectra

For compounds bearing ions with a 3d^0^ configuration, such as V(V), UV-Vis spectra provide information concerning the presence of charge transfer and intraligand transitions. On the other hand, the absence of absorptions at wavelengths higher than 500 nm is a confirmation of the preservation of the oxidation state of the vanadium ion.

The UV–Vis absorption spectrum of ADV displays two well-defined absorption bands at 280 and 350 nm corresponding to the ligand-to-metal charge transfer (LMCT) transitions of the O^2−^→V^5+^ type, as reported in the literature [37]. In the spectra of the (**1**–**4**) DVs (Supplementary Figure S1), both bands are shifted to higher wavenumbers (Table 1). They are accompanied in the UV region by a band assigned to the π→π* transition for guanidinium units. In the spectra of compound (**3**), two well-defined bands appear at 215 and 280 nm due to the two guanidinium derivatives.

#### 2.2.3. Solid-State EPR Spectroscopy

EPR spectroscopy is a highly sensitive technique useful for paramagnetic species with unpaired electron characterization.

**Figure 6:**
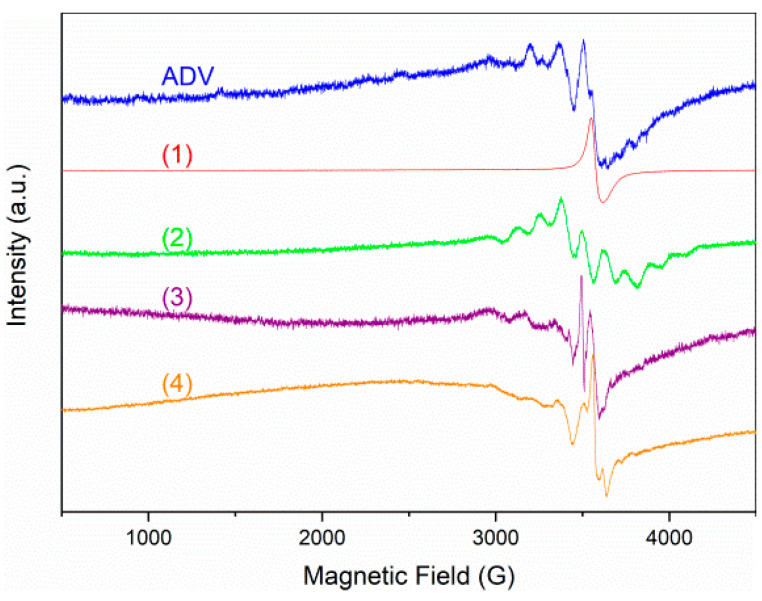
X-band powder EPR spectra of the ADV and (**1**–**4**) compounds.

The solid-state EPR spectra of ADV and the (**1**–**4**) compounds are depicted in Figure 6, showing a resonance with a g-value of 1.97 and a complex hyperfine splitting. Similar EPR spectra are reported in the literature for vanadium-based compounds with a four-oxidation state, which exhibits a characteristic g-factor of 1.968 [38]. Since the V(IV) ion has an electronic spin S = 1/2 and the nuclear spin for the ^51^V isotope (natural abundance 99.5%) is I = 7/2, the isotropic EPR spectrum of isolated V(IV) species consists of a set of eight hyperfine lines due to the dipole–dipole interaction between the magnetic moment of the ^51^V nucleus and the electronic moment of the unpaired V(IV) electron [39]. The EPR spectrum of compound (**1**) presents only one broad EPR line due to strong spin–spin interactions between the paramagnetic centers. The presence of the V(IV) centers is correlated with the charge-transfer process (π(O)→d(V) transition probability) that can occur in the decavanadate units, as observed in the UV-Vis spectra. All these signals disappear through compound solubilization in DMSO.

### 2.3. Antibacterial and Antitumor Activities

#### 2.3.1. Antibacterial Activity

**Figure 7:**
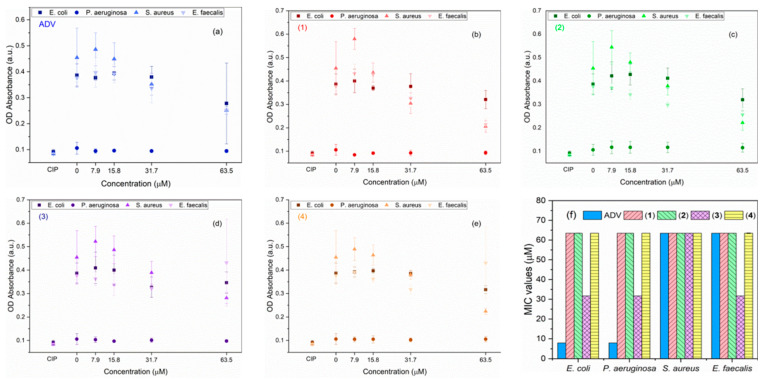
Selected bacteria inhibition by the complexes ADV (**a**), (**1**) (**b**), (**2**) (**c**), (**3**) (**d**), and (**4**) (**e**) at different concentrations, as well as the MIC values (**f**) for the selected bacterial strains.

Based on the literature data, some Gram-negative (*Escherichia coli* and *Pseudomonas aeruginosa*) and Gram-positive (*Staphylococcus aureus* and *Enterococcus faecalis*) bacterial strains with ATCC provenience were chosen for biological assay. The compounds generally inhibited the development of the bacterial strains (Figure 7). *E. coli* and *P. aeruginosa* were most sensitive to ADV and complex (**3**) (the recorded minimal inhibitory concentration (MIC) was 7.9 µM for ADV and 31.7 µM for complex (**3**)). Except for *Enterococcus faecalis* (31.7 µM for (**3**)), all other compounds showed inhibitory abilities at the highest concentration used in this experiment (63.5 µM).

Based on these results, the MIC values were chosen to determine the inhibitory capacity of the compounds in biofilm formation (Supplementary Figure S2). Except for *P. aeruginosa*, all samples exhibited a proliferative response, with registered values over those of the untreated control. This might indicate that either the compounds are not able to disrupt the biofilm in three hours or that the compounds favor biofilm formation if they are left in contact with the bacterial strains for less than 24 h.

Other studies showed that Gram-positive bacteria are more susceptible to ADV than Gram-negative strains [23]. The bacterial wall–ADV interaction is possible due to the membrane potential and charged components. The mechanism of action is explained by several factors: (i) the nutrient uptake from the media is inhibited by the blockage of membrane proteins by ADV [22,25]; (ii) the ADV enters the bacteria and forms intralysosomal decavanadate that could later lead to cellular damage and eventually death [24]; and (iii) decavanadate disrupts the function of some proteins (i.e., ATPases), which later leads to cell death [17,22]. Although probable, these modes of action do not explain completely how ADV inhibits bacterial growth, and further investigations are still required.

#### 2.3.2. Antitumor Activity

**Figure 8:**
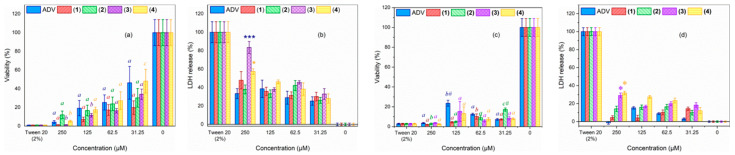
Cytotoxicity assay of the obtained compounds against human normal and cancerous cells; the MTT viability assay (**a**) and LDH release (**b**) of A375 cells; the MTT viability assay (**c**) and LDH release (**d**) of BJ cells; the bars marked with different letters are significantly different from the others between concentrations of the same compound, and those marked with a letter and ‘#’ are significantly different within the same concentration as well (*p* < 0.05 according to the Tukey test); bars marked with ‘*’ are significantly different from the rest according to one way ANOVA (*** *p* < 0.0001, and * *p* < 0.01).

The cytotoxicity was assayed against A 375 human melanoma cells and BJ human fibroblasts by testing the viability by the 3-(4,5-dimethylthiazol-2-yl)-2,5-diphenyltetrazolium bromide (MTT) colorimetric method, lactate dehydrogenase (LDH), and nitric oxide (NO) release. The concentration at which 50% of the cells are affected (IC_50_) was calculated based on the obtained results. All compounds exhibited high cytotoxicity against both melanoma and fibroblast cells (Figure 8a,c), with the best results observed for compound (**1**), followed by (**3**), (**2**), **ADV**, and (**4**) (Table 2). According to the statistical analyses conducted, compounds (**3**) and (**4**) (at 250 µM concentration) had similar toxicity levels as the control treated with Tween 20 against melanoma.

LDH measurements indicate the degree of cellular damage. Thus, the LDH release was observed for all compounds at all tested concentrations (Figure 8b,d), with the highest value registered for compound (**3**) at 250 µM for melanoma and compound (**4**) for fibroblasts.

The tested fibroblasts showed a significant decrease in viability at all concentrations (Figure 8c), while the IC_50_ values determined were similar for all compounds. However, the LDH release registered indicates that the compounds might not affect the external membrane of the BJ cells to the same extent as for A375 cells (Figure 8d).

The NO release in the media was determined to further investigate the degree of induced cytotoxicity. All tested compounds generated NO for at least one concentration of nitrates/nitrites, which could indicate that the vanadium compounds might induce oxidative stress in cancerous cells. However, no changes in the NO level were observed for the BJ cells.

In the case of A375 cells, for every concentration of vanadium compounds at which a low viability was registered, a high LDH concentration was observed in the media. Moreover, NO absorbance indicated nitrites/nitrates in the media at the same concentrations where cell toxicity was detected. Following what was observed herein, vanadium compounds were previously reported to have cytotoxicity in vitro [26,27,28,29,30,31,32,33]. These results indicate that the cells might reach apoptosis through necrosis [40], making the compounds good candidates for potential cancer theranostics. The BJ fibroblasts registered significantly low viability, but as indicated by the other two assays, these values could be associated with either a lower number of cells or a lower number of mitochondria in the cells [41]. Hence, the vanadium compounds might not affect the overall metabolism of normal cells as they do for cancerous cells.

The mechanism of action is dependent on the membrane potential, elasticity, and surface charge, as in prokaryotic cells, which leads to the activation of apoptotic pathways [28]. Since the membrane of tumor cells is more permeable, it can facilitate a higher intake of ADV compared to normal cells [41], similar to other findings [27,29,32].

#### 2.3.3. Solution EPR Spectroscopy

**Figure 9:**
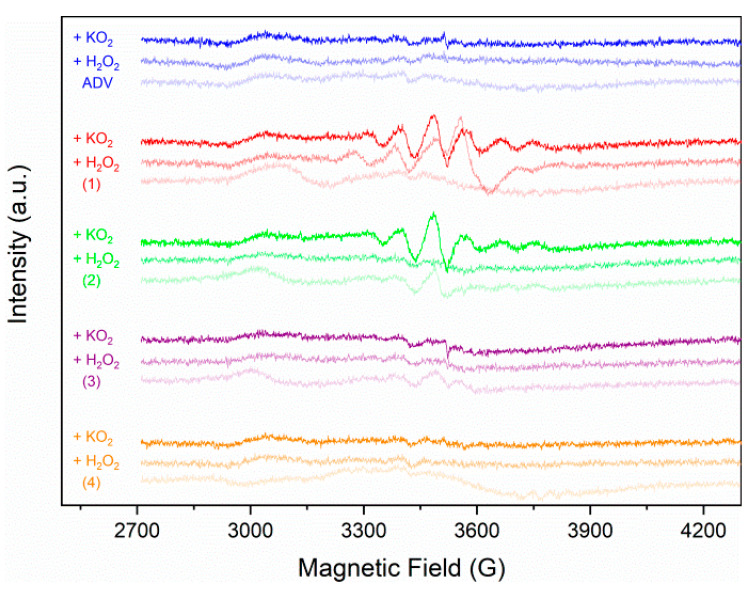
EPR spectra of the ADV and (**1**–**4**) compounds in a 50 mM DMSO solution where KO_2_ and H_2_O_2_ were added as O_2_^−^ and OH^−^ radical donors.

The ability of the proposed DV-based compounds to trap or scavenge reactive oxygen species was tested via EPR spectroscopy, for which KO_2_ and H_2_O_2_ were added as O_2_^−^ and OH^−^ radical donors. The results are presented in Figure 9, showing that compound (**1**) can trap both types of radicals, and thus, the V(IV) characteristic EPR signal appears after adding the radical donors. The same is valid also for compound (**2**), but only for O_2_^−^ radicals. The other compounds, ADV, (**3**), and (4), show no activity at all, indicating that these compounds are not as sensitive in the presence of ROS as (**1**) and (**2**).

### 2.4. Molecular Docking Studies

The cytochrome *bc_1_* complex (complex III) is an essential multi-functional protein located in the inner mitochondrial membrane of eukaryotes or the cytoplasmatic membrane of prokaryotic organisms, being an integral part of the cellular respiratory chain, considering its contribution to the generation of both electrochemical potential and reactive oxygen species [42]. Alteration of the *bc_1_* mechanism by chemical or genetic factors leads to harmful consequences for tissue integrity.

Briefly, the cytochrome *bc_1_* complex is involved in the translocation of protons across the inner mitochondrial membrane due to a joint action of two redox processes between ubiquinol (QH_2_) and ubiquinone (Q), which take place at the distinct catalytic sites located on the opposite side of the lipidic membrane (a quinone reduction site (Q_i_) and a quinol oxidation site (Q_0_)), as well as a third redox process of cytochrome c located in the water phase (intermembrane space or periplasm). An interesting feature of *b*-type cytochrome is that the catalytic activity of the two sites (Q_i_ and Q_0_) is closely related, i.e., the product of one catalytic site becomes the substrate for the second one, and vice versa [43]. When the *bc_1_*-complex is blocked by specific respiratory inhibitors, the electron transfer between cytochrome b and cytochrome c diminishes drastically, and the ROS production is increased, resulting in these species being released into the inter-membrane space or the mitochondrial matrix [44]. Such oxidative stress contributes to the pathogenesis of many diseases.

For this reason, developing drugs (antibiotics, anti-fungal agents, or anti-parasite agents) acting as specific *bc_1_* inhibitors is of commercial interest due to their use in medicine or agriculture to control diseases or cure severe infections. According to the state-of-the-art literature, specific *bc_1_* inhibitors are divided into three classes based on their points of action [45], such as (i) complex III Q_i_ inhibitors (antimycin A, amisulbrom, cyazofamid); (ii) complex III Q_0_ inhibitors (stigmatellin, ametoctradin, azoxystrobin); and (iii) dual site inhibitors (ascochlorin). Moreover, based on in vivo studies, Aureliano et al. [46] pointed out that mitochondria might be a target for DV units that could affect its bioenergetics through different toxicity mechanisms by blocking the respiratory electron transport chain and acting as an antimycin A-like inhibitor.

Our docking study aims to investigate the binding affinities of DV units to the Q_i_ and Q_0_ sites of complex III to predict their activity as mitochondrial inhibitors. It is noteworthy that the structure of cytochrome *bc_1_* presents a dimeric association structure between two identical units, each featuring both Q_i_ and Q_0_ sites. Due to their identical environment, the docking study has been performed on a single set of Q_i_ and Q_0_ sites, as found in chains C or P of the cytochrome *bc_1_*-associated structure.

Regarding the Q_i_ binding pocket located in the C (or P) chain of the enzyme sequence, some key residues were mentioned to be involved in antimycin H-bond interactions (*via* water molecules), namely Asp228, Lys 227, Ser35, His201, and Ser205, in close vicinity of the heme *b_H_*, as reported by Huang et al. [47].

The results of docking calculations indicate that all four DV units were conveniently accommodated inside the Q_i_ site, i.e., in the proximity of the heme *b_H_* with a negative binding energy of −6.29 kcal/mol for the reference ADV compound and for (**1**), (**3**), and (**4**) slightly smaller values (−6.37, −6.31, and −6.3 kcal/mol, respectively), indicating a viable possibility of binding in each case.

**Figure 10:**
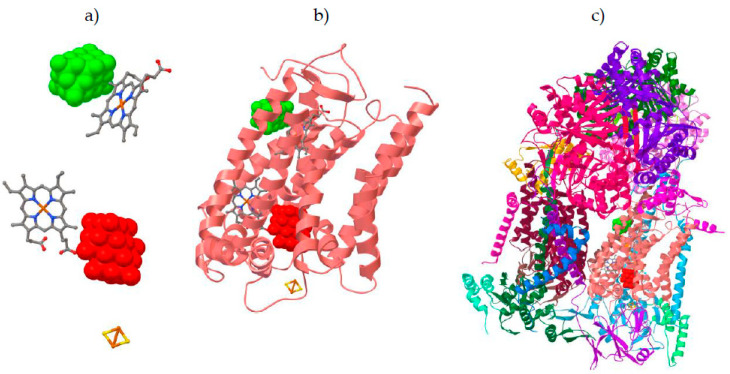
Docking poses of the DV unit in the cytochrome *bc_1_* structure as simulated with the AutoDock-Vina algorithm: successful docking of the DV unit (green) in the Q_i_ site; the forced pose of the DV unit (red) in the Q_0_ site (energetically disfavored docking, see text). Both poses represent a guide for the eye to locate Q_i_ and Q_0_ sites (**a**); a representation of chain C of the enzyme (**b**); and a full representation of the dimeric enzyme structure (**c**). More details can be found in Supplementary Figure S3.

The Q_0_ binding site is located close to both the heme *b_L_* and the Rieske cluster (2Fe-2S cluster, Figure 10) in such an orientation that stigmatellin is positioned between the two prosthetic residues [48] (i.e., it binds to cytochrome *b* at a distal domain from the heme *b_L_*) and interacts with the Rieske cluster through the formation of H bonds with His161, while the Pro270 residue plays an important role in the aromatic π–π interaction [47].

Attempts to dock any DV units into the Q_0_ site failed: negative binding energy was obtained (around −7 kcal/mol) only if the DV unit left the Q_0_ site space, whereas forcing the DV unit to pose in the Q_0_ site near the heme *b_L_* structure leads to unrealistically high (positive) values of the binding energy.

## 3. Materials and Methods

### 3.1. Materials and Physical Measurements

Chemicals were purchased from Sigma-Aldrich (Darmstadt, Germany) (ammonium vanadate, 1-phenyl biguanide 98%, 1-(*o*-tolyl)biguanide 98%, and 1-cyanoguanidine 99%) in reagent grade. All these were used as received without further purification.

Fourier Transform Infrared spectroscopy (FT-IR) spectra were recorded in KBr pellets with a Tensor 37 spectrometer (Bruker, Billerica, MA, USA) in the 400–4000 cm^−1^ range. UV-Vis spectra in solid-state were recorded using a Jasco V 670 spectrophotometer (Jasco, Easton, MD, USA) with Spectralon as a standard in the 200–1500 nm range. Quartz cells measuring 1 cm were used for all measurements. X-band EPR measurements were carried out with a continuous-wave dual-band E500 ELEXSYS EPR spectrometer (Bruker, Karlsruhe, Germany). The room temperature measurements in the X-band were carried out at a microwave frequency of 9.879 GHz. The free radical scavenging ability of the complexes was tested through EPR spectroscopy using KO_2_ and H_2_O_2_ as sources for O_2_^−^ and OH^−^. KO_2_ was dissolved in DMSO by complexation with dibenzo-18-crown-6-ether. The final concentrations used were 500 µM KO_2_ and 3% H_2_O_2_. X-ray diffraction data were collected at 293 K on a Rigaku XtaLAB Synergy-S diffractometer operating with a Mo-Kα (λ = 0.71073 Å) micro-focus sealed X-ray tube. The structure was solved by direct methods and refined using full-matrix least-squares techniques based on F2. The non-H atoms were refined using anisotropic displacement parameters. The calculations were performed using the crystallographic software package SHELX-2018 [49]. The crystallographic data (excluding structure factors) have been deposited with the Cambridge Crystallographic Data Centre with CCDC reference numbers 2303445–2303447. These data can be obtained free of charge from http://www.ccdc.cam.ac.uk/conts/retrieving.html, accessed on 20 October 2023, or from the Cambridge Crystallographic Data Centre, 12 Union Road, Cambridge CB2 1EZ, UK; fax: (+44) 1223-336-033; or e-mail: deposit@ccdc.cam.ac.uk. X-ray powder diffraction measurements (XRPD) were carried out on a D8 Advance Bruker diffractometer (CuKα radiation) equipped with a linear Vantec Super Speed detector and a TTK-450 temperature chamber. Additional XRPD patterns were recorded on a PROTO AXRD Benchtop Powder Diffractometer (CuKα radiation).

### 3.2. Synthesis of Compounds

The compounds (Hpbg)_4_[H_2_V_10_O_28_]·6H_2_O (**1**) and (Htbg)_4_[H_2_V_10_O_28_]·6H_2_O (**2**) were prepared as follows: to a solution containing ammonium vanadate (0.585 g, 5 mmol) in 100 mL of water, a few drops of 37% HCl (pH = 5) were added, and then a solution containing pbg/tbg (0.531/0.573 g, 3 mmol) was dissolved in 50 mL of water. The reaction mixture was magnetically stirred at 50 °C for 1 h and left at room temperature for slow evaporation. Orange crystals were obtained after the solution stood at room temperature for several days, was filtered off, washed several times with cold ethanol, and dried in the air. Yield 86/87%. Anal. Calcd. for C_32_H_62_N_20_O_34_V_10_ (**1**) (%): C, 21.59; H, 3.51; N, 15.73; found (%): C, 21.67; H, 3.38; N, 15.81; calcd. for C_36_H_70_N_20_O_34_V_10_ (**2**) (%): C, 23.65; H, 3.42; N, 15.32; and found (%): C, 23.67; H, 3.32; N, 15.86. The crystals, suitable for X-ray diffraction, were obtained by slow diffusion of an aqueous solution of fbg in an aqueous solution of ADV.

The same solution as for (**2**), completed with 5 mL of hydrogen peroxide at 30%, afforded (Hgnd)_2_(Hgnu)_4_[V_10_O_28_] (**3**) with a yield of 81%. Calcd. for C_10_H_40_N_22_O_32_V_10_ (%): C, 8.06; H, 2.71; N, 20.68; found (%): C, 8.07; H, 2.68; N, 20.71. Crystals suitable for X-ray diffraction were obtained by slow diffusion of an aqueous solution of tbg and H_2_O_2_ 30% in an aqueous solution of ADV.

The compound (Hgdu)_6_[V_10_O_28_]·2H_2_O (**4**) was synthesized by mixing a 100 mL solution containing 0.585 g (5 mmol) of ammonium vanadate in water with a few drops of HCl (37% up to pH 5) and a 50 mL solution with 0.252 g (3 mmol) of cyanoguanidine. The reaction mixture was magnetically stirred at 50 °C for 1 h and left at room temperature for slow evaporation. The orange crystals obtained after the solution stood at room temperature for several days were filtered off, washed several times with cold ethanol, and dried in the air. Yield 89%. Anal. Calcd. for C_12_H_46_N_24_O_36_V_10_ (%): C, 8.94; H, 2.88; N, 20.85; and found (%): C, 8.97; H, 2.85; N, 20.91. X-ray diffraction-suitable crystals were obtained by slow diffusion of an aqueous solution of 1-cyanoguanidine in an aqueous solution of ADV.

All compounds were obtained as crystalline materials. The powder XRD analysis of polycrystalline samples is presented in Supplementary Figure S4. For compound (**2**), several attempts to obtain good diffraction single crystals were unsuccessful.

### 3.3. Antibacterial Assay

The antibacterial properties of the components were assayed against *E. coli* (ATCC 25922), *P. aeruginosa* (ATCC 27853), *S. aureus* (ATCC 25923), and *E. faecalis* (ATCC: 29212) through the microdilution method [50]. Four concentrations were used (7.9, 15.8, 31.7, and 63.5 µM), and the first that registered bacterial inhibition below untreated control or similar to antibiotic control was chosen as the minimal inhibitory concentration (MIC). This concentration was further used to determine the inhibitory capacity of the component on biofilm formation, according to the literature data [51]. Briefly, the samples were incubated with the bacteria (24 h fresh cultures at 1:40 dilution with nutrient media) for 3 h at 37 °C. The solution was removed after this interval and replaced with MTT (1.25 mg/mL final concentration in the well) for 2 h. Later, the MTT solution was removed and replaced with isopropanol for 10 min to dissolve the formazan crystals. The absorbance was read at 550 and 630 nm (Epoch plate reader; BioTek, Bad Friedrichshal, Germany), and the percentage of inhibition was calculated according to the following Equation (1).

%inhibition = (Mean of registered absorbance)/(Mean of untreated control) × 100.(1)

All experiments had four replicates, and the mean was calculated for at least two independent values. One plate containing the components and the media, without bacteria, was also used as a control. A student *t*-test and one-way ANOVA were used to determine the significance level between the compounds applied to one strain.

### 3.4. In Vitro Cytotoxicity Assay

The cytotoxicity was assayed against human melanoma cells (ATCC CRL-1619, Wesel, Germany) and human fibroblasts (BJ, CRL-2522, Wesel, Germany), as previously reported [52]. First, the viability was determined through the MTT test, and the IC_50_ values were calculated. Briefly, the cells inoculated at 12 × 10^3^ cells/well were incubated with the solutions for 24 h at 37 °C. After this, the media was removed and tested for LDH and NO. The wells were incubated with 3-(4,5-dimethylthiazol-2-yl)-2,5-diphenyltetrazolium bromide (MTT, Sigma Aldrich, Merck KGaA, Darmstadt, Germany) for 1.5 h at 37 °C, and the formazan crystals were dissolved with isopropanol. Next, the LDH and NO releases in the media were determined. For LDH, 50 μL lithium lactate solution of 50 mM, 50 μL tris solution of 200 mM, and 50 μL of NAD solution (a mixture of ionitrotetrazolium violet, phenazine metosulfate, and nicotinamide dinucleotide; Sigma Aldrich, Merck KGaA, Darmstadt, Germany) were mixed with 50 µL of media. NO sulphanilamide and N-(-1-napthyl)-ethylenediamine (Sigma Aldrich, Merck KGaA, Darmstadt, Germany) were mixed with 50 µL of media. The plates were read using a BioTek Epoch plate reader (BioTek, Bad Friedrichshal, Germany) and Gen5 software (version 1.09). The concentrations used ranged from 250 to 31.25 µM for all compounds. One-way ANOVA and Tukey statistical analyses were used to determine the significance level within the concentrations and between the compounds.

### 3.5. Computational Strategy

#### Docking Studies

The X-ray diffraction structure of mitochondrial complex III (PDB entry: 1PPJ, deposition on 2003; latest revision in 2017, accessed on 12 March 2023) was obtained from the RCSB Protein Data Bank (https://www.rcsb.org/structure/1PPJ, accessed on 12 March 2023). This crystal structure of bovine heart mitochondrial complex III was chosen for its best resolution (2.1 Å) compared to similar structures reported. Moreover, the co-crystallized ligands (both antimycin A and stigmatellin that also served to identify the location of Q_i_ and Q_0_ sites correctly) were afterward removed (together with all non-proteic residues) from the enzyme structure before initiating the docking procedure. Theoretical calculations were performed using the following software suites: AutoDock-Vina v1.2.3-52 source [53,54] was compiled statically with GCC 8.5.0 using system default boost libs v1.66 and Python Molecule Viewer v1.5.7p1 as implemented in the MGL Tools v.1.5.7 tarball installer [55]. The geometries of the investigated DV units were taken from experimental single crystal measurements without further optimization. Molecular docking calculations were performed with the AutoDock-Vina engine [53,54] using box sizes up to 24 × 24 × 26 Å^3^ and a spacer of 0.375 Å (exhaustiveness parameter 32).

## 4. Conclusions

Several decavanadates bearing guanide moieties were synthesized and characterized. For three species, the structure was determined through single crystal X-ray diffraction. These contain the DV units in interaction with guanidium cations and/or water molecules, generating a supramolecular arrangement through a complex hydrogen bond network. Complex (**3**) was the most active on all planktonic bacterial strains, while compound (**1**) exhibited the highest cytotoxicity on A375 human melanoma cells, this being lower on BJ healthy ones. Both lactate dehydrogenase and nitric oxide-enhanced levels in tumor cells accompanied this activity. Molecular docking calculations predict a specific interaction of the investigated DV units with the Q_i_ site of the cytochrome bc1 complex with very close binding energies, in concordance with the similar order of magnitude of the inhibitory capacity for each bacterial strain case.

## Figures and Tables

**Table 1:** Absorption maxima for ADV and compounds (**1–4**) in UV-Vis spectra.

Compound	Absorption Maxima (nm)	
	π→π*	LMCT
ADV	-	280, 350
(**1**)	260	390, 450
(**2**)	265	395, 425
(**3**)	215, 280	335, 395
(**4**)	265	365, 450

**Table 2:** The IC_50_ values registered for the melanoma and fibroblast cells treated with the vanadium compounds.

Cell	ADV	(1)	(2)	(3)	(4)
	IC_50_ (µM)				
A375	57.5	17.6	39.01	34.6	59.6
BJ	3.63	8.04	4.43	7.21	6.56

## Data Availability

Data are contained within the article and Supplementary Materials.

## Supplementary Materials

The following supporting information can be downloaded at: https://www.mdpi.com/article/10.3390/ijms242417137/s1.
