# Supplementary material for: Decavanadate-Bearing Guanidine Derivatives Developed as Antimicrobial and Antitumor Species
Source: Int J Mol Sci. 2023 Dec 5;24(24):17137. doi: 10.3390/ijms242417137 (PMC10742724; doi:10.3390/ijms242417137)
Supplement: Supplementary file 1 [file ijms-24-17137-s001.zip › ijms-2719105-supplementary.pdf]

Supplementary Material

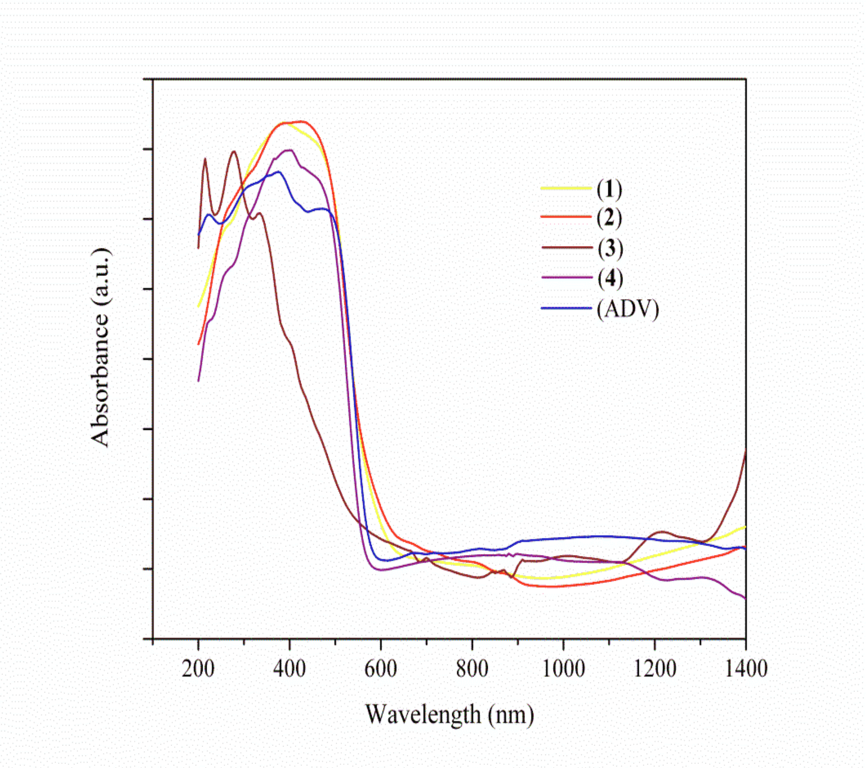

Figure S1. UV-Vis spectra of ADV and complexes (**1**)-(**4**).

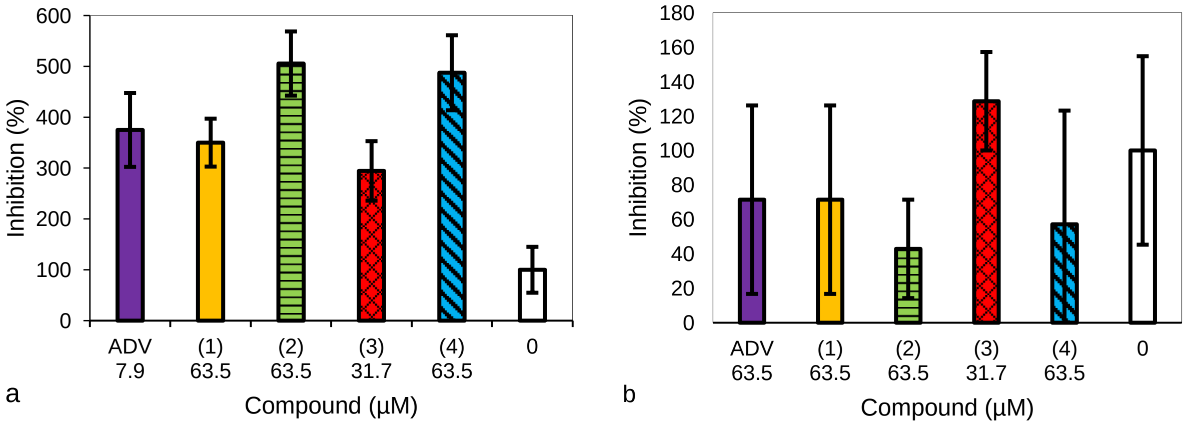

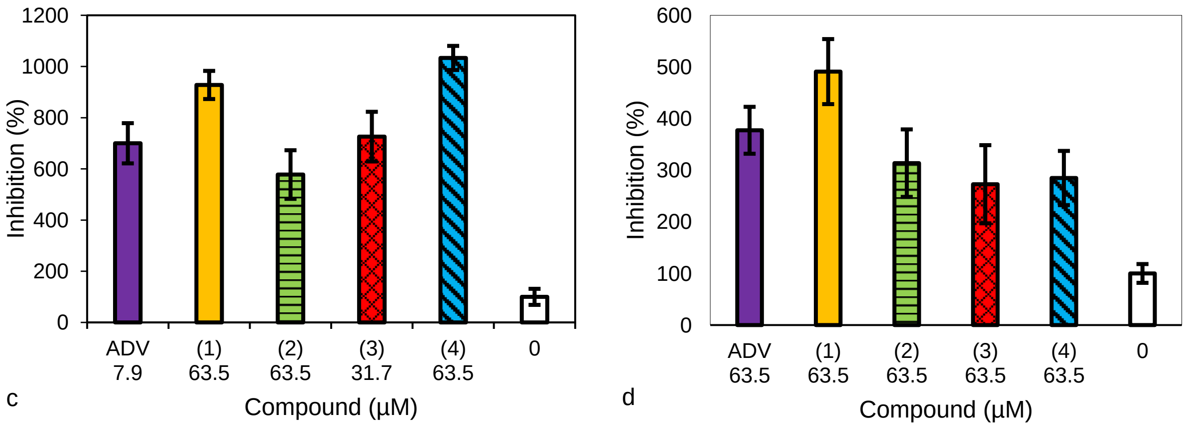

Figure S2. MTT assay showing the inhibitory capacity of the compounds against biofilm formation: *Escherichia coli* (a), *Pseudomonas aeruginosa* (b), *Staphilococcus aureus* (c) and *Enterococcus faecalis* (d).

(a) (b) (c)

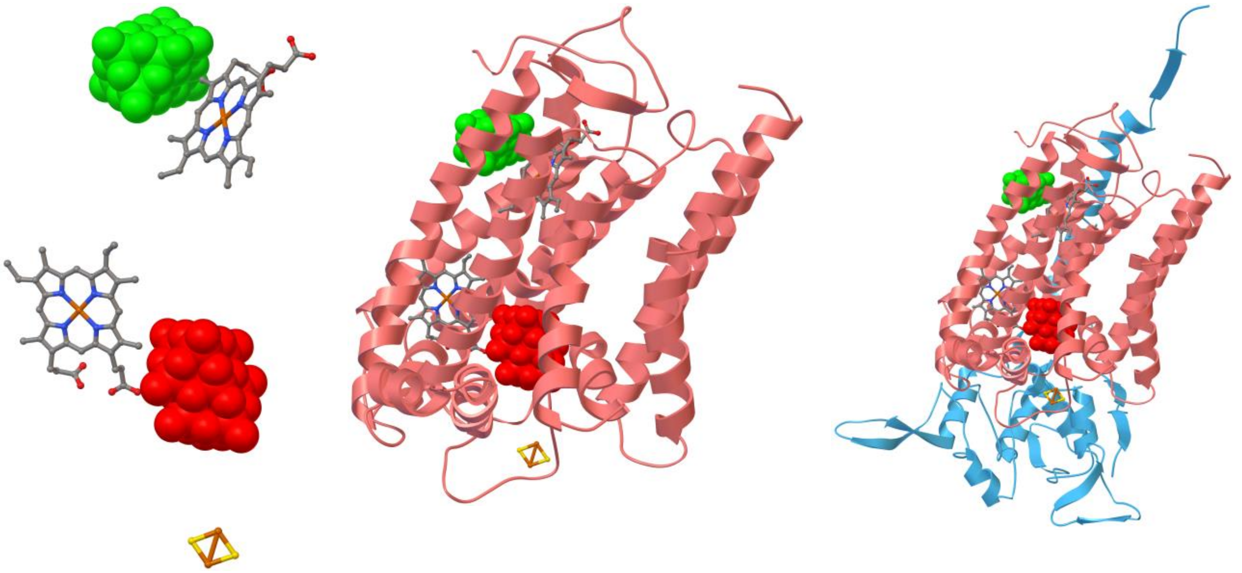

(d) (e) (f)

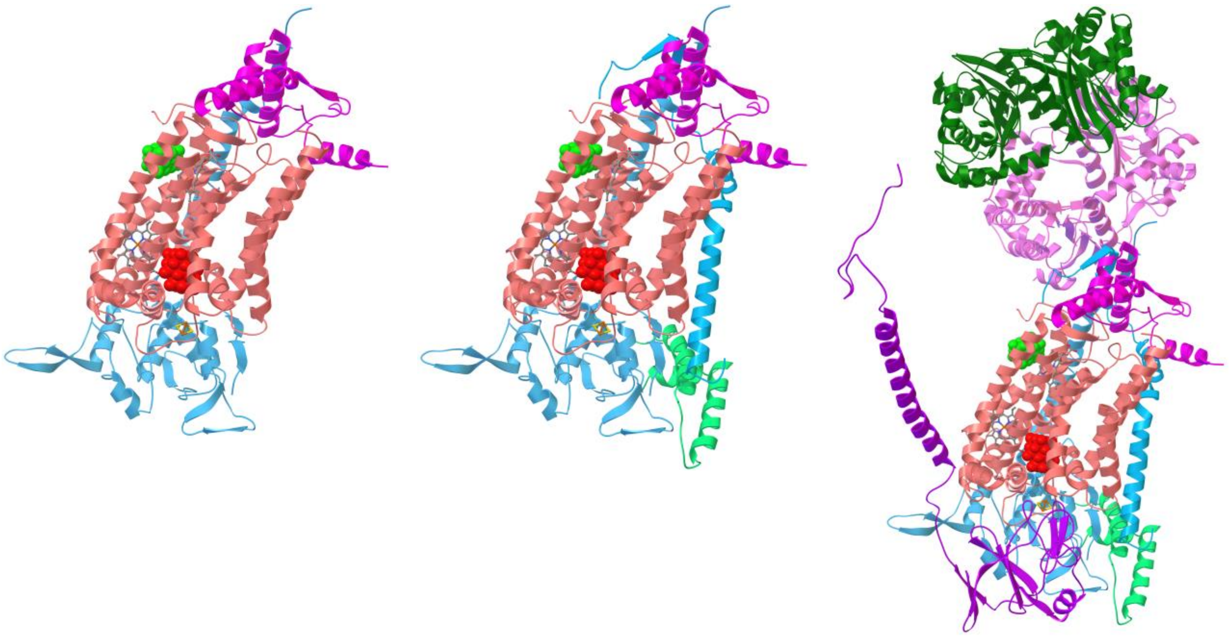

(g)

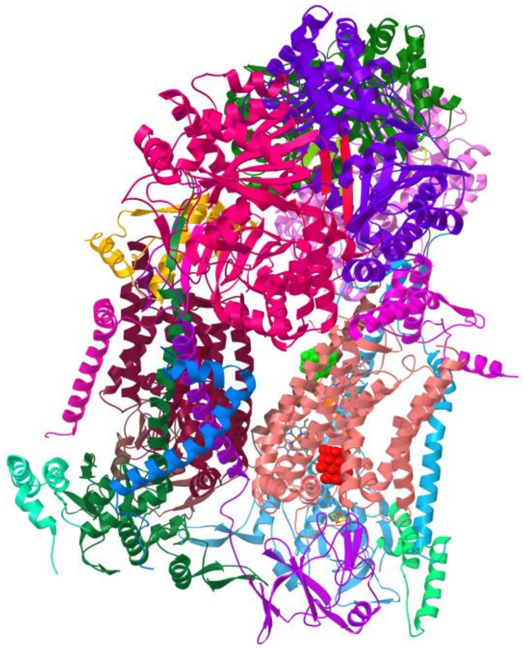

Figure S3. Docking poses of the DV unit in the cytochrome bc1 structure: successful docking of the DV unit (green) in the Q_i site; the forced pose of the DV unit (red) in the Q_0 site (energetically disfavored docking, see text). Both poses were represented as a guide for the eye to locate Q_i and Q_0 sites (a); representation of chain C of the enzyme (b); chain D added to the previous figure (c); chain F added (d); chains G (cyan) and H (green) (**e**); chain A (green), B (violet, top center of the figure) and R (violet, left part) added (f), and full representation of the dimeric enzyme structure (g).

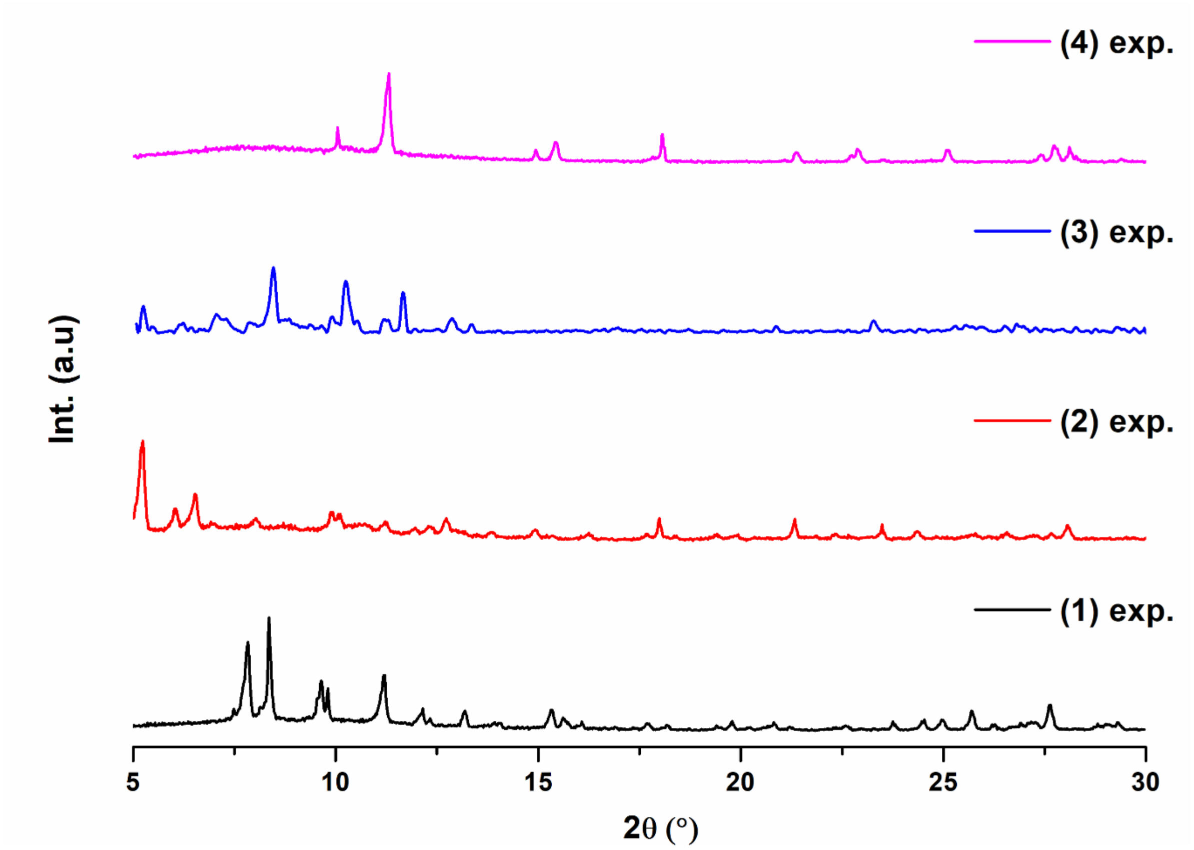

Figure S4. Recorded powder XRD patterns for (**1**)-(**4**).

Table S1. Crystallographic data for compounds (**1**), (**3**) and (**4**).

**Compound**	(**1**)	(**3**)	(**4**)
Formula	$C_{16}H_{31}N_{10}O_{17}V_5$	$C_{10}H_{40}N_{22}O_{32}V_{10}$	$C_6H_{23}N_{12}O_{18}V_5$
$D_{calc.}$/ g cm^{-3}	1.942	2.271	2.211
m/mm^{-1}	1.570	2.167	1.966
Formula Weight	890.21	1490.04	806.06
T/K	293(2)	293(2)	293(2)
Crystal System	triclinic	monoclinic	monoclinic
Space Group	*P*-1	$P2_1/n$	$P2_1/n$
a/Å	11.6583(5)	11.5479(5)	13.4573(6)
b/Å	12.0095(5)	13.9485(4)	14.2365(4)
c/Å	12.6169(4)	14.3688(6)	14.0297(7)
a/°	114.763(3)	90	90
b/°	95.903(3)	109.690(4)	115.723(6)
g/°	103.146(3)	90	90
V/Å^3	1522.33(10)	2179.14(15)	2421.5(2)
Z	2	2	4
Z'	1	0.5	1
Wavelength/Å	0.71073	0.71073	0.71073
Radiation type	MoK$_a$	MoK$_a$	MoK$_a$
Q_{min}/°	1.957	2.097	2.155
Q_{max}/°	30.942	30.724	30.780
Measured Refl.	18773	15664	17522
Independent Refl.	7338	5262	5835
Reflections with I > 2(I)	6023	4204	4834
R_{int}	0.0312	0.0537	0.0349
Parameters	429	334	377
Restraints	10	0	3
Largest Peak	0.638	0.760	0.398
Deepest Hole	-0.501	-0.740	-0.424
GooF	1.061	1.057	1.081
wR_2 (all data)	0.0954	0.1145	0.1001
wR_2	0.0893	0.1044	0.0941
R_1 (all data)	0.0455	0.0557	0.0413
R_1	0.0361	0.0412	0.0322

Table S2. Bond lengths in Å for (**1**).

**Atom**	**Atom**	**Length/Å**	**Atom**	**Atom**	**Length/Å**
V1	V5	3.0872(5)	N20	C19	1.328(4)

Atom	Atom	Length/Å	Atom	Atom	Length/Å
V1	O6	2.0208(15)	C29	C34	1.374(4)
V1	O7	1.6943(15)	C29	N35	1.435(3)
V1	O23	2.1134(15)	C29	C30	1.390(3)
V1	O23[1]	2.0808(14)	C34	C33	1.387(4)
V1	O25	1.6804(16)	N35	C36	1.325(3)
V1	O28	1.8997(15)	C32	C33	1.383(4)
V2	O6	2.1144(15)	C32	C31	1.373(4)
V2	O23[1]	2.2722(14)	C30	C31	1.385(4)
V2	O26	1.8258(16)	N41	C36	1.326(3)
V2	O27	1.6175(16)	N39	C38	1.319(3)
V2	O28[1]	1.9982(15)	N22	C17	1.328(3)
V2	O46	1.7622(15)	N16	C17	1.360(3)
V3	V4	3.0908(5)	N16	C10	1.425(3)
V3	V5	3.0983(5)	C19	N18	1.346(3)
V3	O6[1]	2.1043(16)	C19	N21	1.330(4)
V3	O8	1.7701(16)	N18	C17	1.329(3)
V3	O9	1.8333(16)	C11	C10	1.383(4)
V3	O23[1]	2.2684(14)	C11	C12	1.385(4)
V3	O28	2.0064(14)	C15	C14	1.387(5)
V3	O47	1.6100(16)	C15	C10	1.393(4)
V4	V5	3.0691(6)	C14	C13	1.379(5)
V4	O7[1]	2.0524(16)	C13	C12	1.369(5)
V4	O9	1.8321(16)	N37	C36	1.361(3)
V4	O23[1]	2.3609(14)	N37	C38	1.350(3)
V4	O24	1.8328(16)	N40	C38	1.339(3)
V4	O45	1.6005(16)	N20	C19	1.328(4)
V4	O46	1.9416(16)	C29	C34	1.374(4)
V5	O8	1.9164(16)	C29	N35	1.435(3)
V5	O23[1]	2.3470(15)	C29	C30	1.390(3)
V5	O24	1.8306(16)	C34	C33	1.387(4)
V5	O25	2.0798(16)	N35	C36	1.325(3)
V5	O26	1.8644(16)	C32	C33	1.383(4)
V5	O44	1.5921(18)	C32	C31	1.373(4)

[1]-x,-y,-z

Table S3. Bond angles in ° for (**1**).

Atom	Atom	Atom	Angle/°	Atom	Atom	Atom	Angle/°
O6	V1	V5	88.73(4)	O23^{1}	V3	V5	48.92(4)
O6	V1	O23	80.06(6)	O28	V3	V4	124.23(5)
O6	V1	O23^{1}	80.83(6)	O28	V3	V5	89.20(4)
O7	V1	V5	146.07(6)	O28	V3	O6^{1}	74.10(6)
O7	V1	O6	93.84(7)	O28	V3	O23^{1}	74.97(6)
O7	V1	O23^{1}	163.99(7)	O47	V3	V4	135.91(6)
O7	V1	O23	86.40(7)	O47	V3	V5	138.65(7)
O7	V1	O28	98.74(7)	O47	V3	O6^{1}	97.32(8)
O23^{1}	V1	V5	49.45(4)	O47	V3	O8	104.54(9)
O23	V1	V5	127.25(4)	O47	V3	O9	103.55(8)
O23^{1}	V1	O23	77.84(6)	O47	V3	O23^{1}	170.92(7)
O25	V1	V5	39.30(5)	O47	V3	O28	98.70(8)
O25	V1	O6	95.74(7)	V5	V4	V3	60.393(12)
O25	V1	O7	106.87(8)	O7^{1}	V4	V3	83.36(5)
O25	V1	O23	166.40(7)	O7^{1}	V4	V5	121.67(4)
O25	V1	O23^{1}	88.75(7)	O7^{1}	V4	O23^{1}	72.59(6)
O25	V1	O28	99.08(7)	O9	V4	V3	32.53(5)
O28	V1	V5	91.52(5)	O9	V4	V5	85.57(5)
O28	V1	O6	156.84(7)	O9	V4	O7^{1}	84.99(7)
O28	V1	O23	81.40(6)	O9	V4	O23^{1}	78.42(6)
O28	V1	O23^{1}	81.80(6)	O9	V4	O24	93.93(7)
O6	V2	O23^{1}	74.55(5)	O9	V4	O46	154.12(7)
O26	V2	O6	86.24(6)	O23^{1}	V4	V3	46.85(4)
O26	V2	O23^{1}	79.82(6)	O23^{1}	V4	V5	49.12(4)
O26	V2	O28^{1}	151.65(7)	O24	V4	V3	82.22(5)
O27	V2	O6	98.14(7)	O24	V4	V5	33.07(5)
O27	V2	O23^{1}	172.18(7)	O24	V4	O7^{1}	154.45(7)
O27	V2	O26	102.77(8)	O24	V4	O23^{1}	82.16(6)
O27	V2	O28^{1}	100.07(8)	O24	V4	O46	89.31(7)
O27	V2	O46	104.34(8)	O45	V4	V3	136.30(7)
O28^{1}	V2	O6	74.04(6)	O45	V4	V5	138.05(7)
O28^{1}	V2	O23^{1}	75.46(6)	O45	V4	O7^{1}	100.01(8)
O46	V2	O6	155.75(7)	O45	V4	O9	103.91(8)
O46	V2	O23^{1}	82.48(6)	O45	V4	O23^{1}	172.16(8)
O46	V2	O26	97.41(7)	O45	V4	O24	104.99(9)
O46	V2	O28^{1}	92.95(7)	O45	V4	O46	99.99(8)
V4	V3	V5	59.456(12)	O46	V4	V3	123.42(5)
O6^{1}	V3	V4	86.42(4)	O46	V4	V5	83.25(5)
O6^{1}	V3	V5	123.75(4)	O46	V4	O7^{1}	81.21(6)
O6^{1}	V3	O23^{1}	74.83(6)	O46	V4	O23^{1}	76.61(6)
O8	V3	V4	85.26(5)	V1	V5	V3	60.908(11)
O8	V3	V5	34.31(5)	V4	V5	V1	91.819(14)
O8	V3	O6^{1}	155.67(7)	V4	V5	V3	60.151(12)
O8	V3	O9	97.78(7)	O8	V5	V1	77.06(5)
O8	V3	O23^{1}	82.42(7)	O8	V5	V3	31.38(5)
O8	V3	O28	91.94(7)	O8	V5	V4	83.61(5)
O9	V3	V4	32.50(5)	O8	V5	O23^{1}	77.41(6)
O9	V3	V5	84.68(5)	O8	V5	O25	81.41(6)
O9	V3	O6^{1}	87.17(7)	O23^{1}	V5	V1	42.35(3)
O9	V3	O23^{1}	80.92(6)	O23^{1}	V5	V3	46.77(4)
O9	V3	O28	152.54(7)	O23^{1}	V5	V4	49.51(3)
O23^{1}	V3	V4	49.40(4)	O24	V5	V1	124.93(5)

Table S4. Bond lengths in Å for (**3**).

Atom	Atom	Length/Å	Atom	Atom	Length/Å
V1	O14	1.690(2)	V4	O16	1.871(2)
V1	O10	1.694(2)	V4	O13	1.887(2)
V1	O7	1.9132(19)	V4	O10[1]	2.039(2)
V1	O6	1.9357(19)	V4	O8	2.3351(18)
V1	O8	2.0963(18)	V4	V5	3.0717(6)
V1	O8[1]	2.1298(19)	V5	O18	1.598(2)
V1	V5	3.0712(7)	V5	O12	1.8318(19)
V1	V4[1]	3.0902(7)	V5	O17	1.874(2)
V2	O11	1.6216(19)	V5	O9	1.896(2)
V2	O17	1.815(2)	V5	O14	2.052(2)
V2	O16	1.828(2)	V5	O8	2.3179(19)
V2	O6[1]	2.010(2)	O2	C3	1.230(4)
V2	O7	2.0122(19)	O1	C5	1.225(4)
V2	O8	2.2264(18)	N6	C2	1.370(4)
V2	V3[1]	3.0867(7)	N6	C3	1.399(4)
V2	V4	3.0951(7)	N11	C4	1.367(4)
V2	V5	3.0961(6)	N11	C5	1.419(4)
V3	O15	1.6198(19)	N4	C2	1.306(4)
V3	O9	1.808(2)	N2	C1	1.328(4)
V3	O13	1.8293(19)	N1	C1	1.319(4)
V3	O6	1.9965(19)	N3	C1	1.330(4)
V3	O7[1]	2.019(2)	N12	C5	1.326(4)
V3	O8	2.2281(18)	N5	C2	1.321(4)
V3	V5	3.0940(7)	N10	C4	1.323(4)
V4	O26	1.597(2)	N9	C4	1.313(4)
V4	O12	1.851(2)	N7	C3	1.324(4)

[1]-x,-y,-z

Table S5. Bond Angles in ° for (**3**).

Atom	Atom	Atom	Angle/°
O14	V1	O10	107.09(10)
O14	V1	O7	98.36(9)
O10	V1	O7	97.28(9)
O14	V1	O6	96.78(9)
O10	V1	O6	95.83(9)
O7	V1	O6	156.09(8)
O14	V1	O8	87.86(8)
O10	V1	O8	164.97(9)
O7	V1	O8	81.71(7)
O6	V1	O8	80.47(7)
O14	V1	O8[1]	165.89(9)
O10	V1	O8[1]	86.91(8)
O7	V1	O8[1]	81.22(8)
O6	V1	O8[1]	79.61(8)
O8	V1	O8[1]	78.11(8)
O14	V1	V5	38.91(7)
O10	V1	V5	145.99(7)
O7	V1	V5	90.46(6)
O6	V1	V5	89.77(6)
O8	V1	V5	48.96(5)
O8[1]	V1	V5	127.06(5)
O14	V1	V4[1]	144.96(7)
O10	V1	V4[1]	37.89(7)
O7	V1	V4[1]	89.87(6)
O6	V1	V4[1]	88.29(6)
O8	V1	V4[1]	127.12(5)
O8[1]	V1	V4[1]	49.02(5)
V5	V1	V4[1]	175.93(2)
O11	V2	O17	104.42(10)
O11	V2	O16	101.43(9)
O17	V2	O16	95.46(9)
O11	V2	O6[1]	98.00(9)
O17	V2	O6[1]	155.43(8)
O16	V2	O6[1]	89.76(8)
O11	V2	O7	99.25(9)
O17	V2	O7	90.13(8)
O16	V2	O7	156.47(9)
O6[1]	V2	O7	76.31(8)
O11	V2	O8	173.02(9)
O17	V2	O8	81.22(8)
O16	V2	O8	81.88(8)
O6[1]	V2	O8	75.76(7)
O7	V2	O8	76.40(7)
O11	V2	V3[1]	88.78(7)
O17	V2	V3[1]	130.21(6)
O16	V2	V3[1]	129.21(7)
O6[1]	V2	V3[1]	39.45(5)
O7	V2	V3[1]	40.10(5)

Atom	Atom	Atom	Angle/°
O8	V2	V3[1]	84.39(5)
O11	V2	V4	135.02(7)
O17	V2	V4	84.38(6)
O16	V2	V4	33.63(6)
O6[1]	V2	V4	86.86(6)
O7	V2	V4	125.12(6)
O8	V2	V4	48.77(5)
V3[1]	V2	V4	119.23(2)
O11	V2	V5	137.74(8)
O17	V2	V5	33.54(6)
O16	V2	V5	84.28(6)
O6[1]	V2	V5	124.05(6)
O7	V2	V5	87.94(5)
O8	V2	V5	48.31(5)
V3[1]	V2	V5	119.991(18)
V4	V2	V5	59.490(15)
O15	V3	O9	102.81(10)
O15	V3	O13	102.98(10)
O9	V3	O13	95.52(9)
O15	V3	O6	99.31(9)
O9	V3	O6	90.97(9)
O13	V3	O6	154.74(8)
O15	V3	O7[1]	97.99(9)
O9	V3	O7[1]	157.22(9)
O13	V3	O7[1]	88.71(8)
O6	V3	O7[1]	76.47(8)
O15	V3	O8	173.45(9)
O9	V3	O8	82.01(8)
O13	V3	O8	80.76(8)
O6	V3	O8	76.00(7)
O7[1]	V3	O8	76.58(7)
O15	V3	V2[1]	88.79(7)
O9	V3	V2[1]	130.75(7)
O13	V3	V2[1]	128.64(6)
O6	V3	V2[1]	39.78(6)
O7[1]	V3	V2[1]	39.95(5)
O8	V3	V2[1]	84.69(5)
O15	V3	V5	136.88(8)
O9	V3	V5	34.23(6)
O13	V3	V5	83.84(7)
O6	V3	V5	88.02(6)
O7[1]	V3	V5	124.92(6)
O8	V3	V5	48.34(5)
V2[1]	V3	V5	120.254(19)
O26	V4	O12	105.53(10)
O26	V4	O16	100.33(9)
O12	V4	O16	92.49(9)
O26	V4	O13	103.79(10)
O12	V4	O13	89.84(9)
O16	V4	O13	154.18(9)
O26	V4	O10[1]	98.60(9)
O12	V4	O10[1]	155.83(8)
O16	V4	O10[1]	84.42(9)
O13	V4	O10[1]	83.03(8)
O26	V4	O8	172.70(9)

Atom	Atom	Atom	Angle/°
O12	V4	O8	81.71(8)
O16	V4	O8	78.09(7)
O13	V4	O8	76.81(7)
O10[1]	V4	O8	74.19(7)
O26	V4	V5	138.76(8)
O12	V4	V5	33.28(6)
O16	V4	V5	84.33(6)
O13	V4	V5	83.61(6)
O10[1]	V4	V5	122.64(6)
O8	V4	V5	48.45(5)
O26	V4	V1[1]	129.27(8)
O12	V4	V1[1]	125.19(6)
O16	V4	V1[1]	79.88(6)
O13	V4	V1[1]	77.81(6)
O10[1]	V4	V1[1]	30.68(5)
O8	V4	V1[1]	43.51(5)
V5	V4	V1[1]	91.958(17)
O26	V4	V2	133.09(8)
O12	V4	V2	82.64(6)
O16	V4	V2	32.77(6)
O13	V4	V2	122.62(6)
O10[1]	V4	V2	81.90(6)
O8	V4	V2	45.81(4)
V5	V4	V2	60.272(15)
V1[1]	V4	V2	62.094(15)
O18	V5	O12	102.18(10)
O18	V5	O17	102.79(10)
O12	V5	O17	92.69(8)
O18	V5	O9	101.14(10)
O12	V5	O9	90.84(9)
O17	V5	O9	154.47(9)
O18	V5	O14	101.06(10)
O12	V5	O14	156.71(9)
O17	V5	O14	83.56(8)
O9	V5	O14	83.27(8)
O18	V5	O8	175.17(9)
O12	V5	O8	82.59(8)
O17	V5	O8	77.59(8)
O9	V5	O8	77.82(8)
O14	V5	O8	74.15(7)
O18	V5	V1	132.21(8)
O12	V5	V1	125.60(7)
O17	V5	V1	78.25(6)
O9	V5	V1	79.16(6)
O14	V5	V1	31.15(5)
O8	V5	V1	43.01(4)
O18	V5	V4	135.85(8)
O12	V5	V4	33.68(6)
O17	V5	V4	84.14(6)
O9	V5	V4	84.88(6)
O14	V5	V4	123.08(6)
O8	V5	V4	48.93(4)
V1	V5	V4	91.939(17)
O18	V5	V3	133.47(8)
O12	V5	V3	81.77(6)

Atom	Atom	Atom	Angle/°
O17	V5	V3	123.49(7)
O9	V5	V3	32.45(6)
O14	V5	V3	81.25(6)
O8	V5	V3	45.90(4)
V1	V5	V3	61.594(15)
V4	V5	V3	60.996(15)
O18	V5	V2	135.00(8)
O12	V5	V2	82.90(6)
O17	V5	V2	32.35(6)
O9	V5	V2	123.65(6)
O14	V5	V2	81.74(5)
O8	V5	V2	45.83(4)
V1	V5	V2	61.141(15)
V4	V5	V2	60.238(15)
V3	V5	V2	91.507(17)
V1	O6	V3	106.78(9)
V1	O6	V2[1]	107.87(9)
V3	O6	V2[1]	100.77(8)
V1	O7	V2	106.06(9)
V1	O7	V3[1]	106.91(9)
V2	O7	V3[1]	99.95(8)
V1	O8	V1[1]	101.89(8)
V1	O8	V2	92.99(7)
V1[1]	O8	V2	94.13(7)
V1	O8	V3	93.72(7)
V1[1]	O8	V3	92.91(7)
V2	O8	V3	169.07(10)
V1	O8	V5	88.02(6)
V1[1]	O8	V5	170.06(9)
V2	O8	V5	85.87(7)
V3	O8	V5	85.76(6)
V1	O8	V4	170.60(10)
V1[1]	O8	V4	87.47(6)
V2	O8	V4	85.42(6)
V3	O8	V4	86.56(6)
V5	O8	V4	82.62(6)
V3	O9	V5	113.32(10)
V1	O10	V4[1]	111.43(10)
V5	O12	V4	113.04(10)
V3	O13	V4	114.71(10)
V1	O14	V5	109.95(9)
V2	O16	V4	113.60(10)
V2	O17	V5	114.11(10)
C2	N6	C3	125.0(2)
C4	N11	C5	124.9(3)
N4	C2	N5	121.4(3)
N4	C2	N6	121.5(3)
N5	C2	N6	117.1(3)
N1	C1	N2	120.1(3)
N1	C1	N3	120.3(3)
N2	C1	N3	119.6(3)
O2	C3	N7	124.3(3)
O2	C3	N6	121.8(3)
N7	C3	N6	113.8(3)
O1	C5	N12	125.1(3)
O1	C5	N11	121.4(3)
N12	C5	N11	113.4(3)
N9	C4	N10	120.8(3)
N9	C4	N11	121.4(3)
N10	C4	N11	117.8(3)

[1] 1-x,1-y,1-z

Table S6. Bond lengths in Å for (**4**).

Atom	Atom	Length/Å
V1	O10	1.6836(16)
V1	O9	1.6997(17)
V1	O7	1.9269(17)
V1	O6	1.9305(16)
V1	O8	2.0721(16)
V1	O8[1]	2.1335(15)
V1	V5	3.0510(5)
V1	V4[1]	3.0748(5)
V2	O16	1.6106(18)
V2	O12	1.8075(16)
V2	O17	1.8326(17)
V2	O7	2.0114(17)
V2	O6[1]	2.0336(15)
V2	O8	2.2227(16)
V2	V3[1]	3.0857(5)
V2	V4	3.1073(6)
V3	O15	1.6048(18)
V3	O13	1.8153(16)
V3	O11	1.8322(17)
V3	O6	1.9834(17)
V3	O7[1]	2.0207(16)
V3	O8	2.2794(16)
V3	V5	3.0898(5)
V4	O18	1.6088(18)
V4	O14	1.8320(16)
V4	O11	1.8591(17)
V4	O17	1.8889(17)
V4	O9[1]	2.0261(16)
V4	O8	2.3078(16)
V4	V5	3.0657(6)
V5	O19	1.5924(17)
V5	O14	1.8374(17)
V5	O13	1.8375(17)
V5	O12	1.9315(18)
V5	O10	2.0614(18)
V5	O8	2.3201(15)
O20	C21	1.233(3)
O27	C28	1.224(3)
N22	C23	1.360(4)
N22	C21	1.409(3)
O34	C35	1.230(3)
N29	C30	1.366(3)
N29	C28	1.394(4)
N36	C37	1.360(3)
N36	C35	1.391(4)
N31	C30	1.302(3)
N32	C30	1.316(3)
N38	C37	1.310(3)
N40	C35	1.328(4)
N26	C21	1.317(4)
N33	C28	1.324(4)
N24	C23	1.304(3)
N39	C37	1.317(4)
C23	N25	1.299(4)

1 ——

1 [1]1-x,1-y,1-z

1

Table S7. Bond angles in ° for (**4**).

Atom	Atom	Atom	Angle/°
O10	V1	O9	105.89(8)
O10	V1	O7	97.66(8)
O9	V1	O7	96.38(8)
O10	V1	O6	98.08(8)
O9	V1	O6	95.62(8)
O7	V1	O6	156.76(7)
O10	V1	O8	89.22(7)
O9	V1	O8	164.88(7)
O7	V1	O8	81.93(7)
O6	V1	O8	81.30(7)
O10	V1	O8[1]	167.55(8)
O9	V1	O8[1]	86.55(7)
O7	V1	O8[1]	81.19(6)
O6	V1	O8[1]	79.76(6)
O8	V1	O8[1]	78.34(7)
O10	V1	V5	39.79(6)
O9	V1	V5	145.61(5)
O7	V1	V5	91.95(5)
O6	V1	V5	89.29(5)
O8	V1	V5	49.45(4)
O8[1]	V1	V5	127.76(5)
O10	V1	V4[1]	143.87(6)
O9	V1	V4[1]	37.99(5)
O7	V1	V4[1]	88.40(5)
O6	V1	V4[1]	88.91(5)
O8	V1	V4[1]	126.91(4)
O8[1]	V1	V4[1]	48.58(4)
V5	V1	V4[1]	176.201(16)
O16	V2	O12	102.85(8)
O16	V2	O17	102.49(9)
O12	V2	O17	95.98(8)
O16	V2	O7	98.91(8)
O12	V2	O7	90.86(7)
O17	V2	O7	155.43(7)

Atom	Atom	Atom	Angle/°
O16	V2	O6[1]	99.20(8)
O12	V2	O6[1]	155.78(7)
O17	V2	O6[1]	88.87(7)
O7	V2	O6[1]	75.84(6)
O16	V2	O8	173.58(8)
O12	V2	O8	81.79(7)
O17	V2	O8	81.24(7)
O7	V2	O8	76.41(6)
O6[1]	V2	O8	75.51(6)
O16	V2	V3[1]	88.77(7)
O12	V2	V3[1]	131.04(6)
O17	V2	V3[1]	128.08(5)
O7	V2	V3[1]	40.18(5)
O6[1]	V2	V3[1]	39.22(5)
O8	V2	V3[1]	84.82(4)
O16	V2	V4	136.34(7)
O12	V2	V4	84.75(5)
O17	V2	V4	33.96(5)
O7	V2	V4	124.18(5)
O6[1]	V2	V4	86.21(5)
O8	V2	V4	47.85(4)
V3[1]	V2	V4	118.583(15)
O15	V3	O13	104.20(9)
O15	V3	O11	103.71(9)
O13	V3	O11	94.79(7)
O15	V3	O6	100.71(8)
O13	V3	O6	89.96(7)
O11	V3	O6	153.11(7)
O15	V3	O7[1]	99.36(8)
O13	V3	O7[1]	154.75(7)
O11	V3	O7[1]	88.26(7)
O6	V3	O7[1]	76.75(6)
O15	V3	O8	174.09(8)
O13	V3	O8	80.20(7)
O11	V3	O8	79.62(7)
O6	V3	O8	75.14(6)
O7[1]	V3	O8	75.71(6)
O15	V3	V2[1]	89.88(7)
O13	V3	V2[1]	130.35(6)
O11	V3	V2[1]	128.19(5)
O6	V3	V2[1]	40.41(4)
O7[1]	V3	V2[1]	39.96(5)
O8	V3	V2[1]	84.24(4)
O15	V3	V5	136.42(7)
O13	V3	V5	32.46(6)
O11	V3	V5	82.84(5)
O6	V3	V5	87.23(4)
O7[1]	V3	V5	124.07(5)
O8	V3	V5	48.36(4)
V2[1]	V3	V5	120.091(16)
O18	V4	O14	104.85(8)
O18	V4	O11	101.10(9)
O14	V4	O11	91.16(7)
O18	V4	O17	101.91(9)
O14	V4	O17	90.73(7)

Atom	Atom	Atom	Angle/°
O11	V4	O17	155.60(8)
O18	V4	O9[1]	98.16(8)
O14	V4	O9[1]	156.98(7)
O11	V4	O9[1]	85.10(7)
O17	V4	O9[1]	83.71(7)
O18	V4	O8	173.11(7)
O14	V4	O8	82.04(7)
O11	V4	O8	78.34(7)
O17	V4	O8	77.84(7)
O9[1]	V4	O8	74.96(6)
O18	V4	V5	138.20(7)
O14	V4	V5	33.39(5)
O11	V4	V5	83.13(5)
O17	V4	V5	85.14(5)
O9[1]	V4	V5	123.64(5)
O8	V4	V5	48.68(4)
O18	V4	V1[1]	129.23(6)
O14	V4	V1[1]	125.92(6)
O11	V4	V1[1]	79.45(5)
O17	V4	V1[1]	79.78(5)
O9[1]	V4	V1[1]	31.09(5)
O8	V4	V1[1]	43.88(4)
V5	V4	V1[1]	92.550(15)
O18	V4	V2	134.70(7)
O14	V4	V2	81.27(5)
O11	V4	V2	123.88(5)
O17	V4	V2	32.82(5)
O9[1]	V4	V2	82.04(5)
O8	V4	V2	45.57(4)
V5	V4	V2	60.840(13)
V1[1]	V4	V2	62.379(12)
O19	V5	O14	104.43(9)
O19	V5	O13	104.33(9)
O14	V5	O13	93.41(8)
O19	V5	O12	99.35(9)
O14	V5	O12	90.02(8)
O13	V5	O12	154.38(7)
O19	V5	O10	99.54(9)
O14	V5	O10	155.66(7)
O13	V5	O10	84.62(7)
O12	V5	O10	81.92(7)
O19	V5	O8	172.96(8)
O14	V5	O8	81.58(6)
O13	V5	O8	78.65(6)
O12	V5	O8	76.75(6)
O10	V5	O8	74.23(6)
O19	V5	V1	131.05(7)
O14	V5	V1	124.31(5)
O13	V5	V1	79.05(5)
O12	V5	V1	78.08(5)
O10	V5	V1	31.52(4)
O8	V5	V1	42.74(4)
O19	V5	V4	137.70(8)
O14	V5	V4	33.28(5)
O13	V5	V4	85.03(5)

Atom	Atom	Atom	Angle/°
O12	V5	V4	84.01(5)
O10	V5	V4	122.56(5)
O8	V5	V4	48.34(4)
V1	V5	V4	91.079(14)
O19	V5	V3	136.27(8)
O14	V5	V3	82.92(5)
O13	V5	V3	32.02(5)
O12	V5	V3	123.99(5)
O10	V5	V3	82.69(5)
O8	V5	V3	47.24(4)
V1	V5	V3	61.897(12)
V4	V5	V3	60.915(13)
V1	O6	V3	107.58(8)
V1	O6	V2[1]	107.71(7)
V3	O6	V2[1]	100.37(7)
V1	O7	V2	105.13(7)
V1	O7	V3[1]	108.23(7)
V2	O7	V3[1]	99.87(7)
V1	O8	V1[1]	101.66(7)
V1	O8	V2	93.40(6)
V1[1]	O8	V2	94.59(6)
V1	O8	V3	92.94(6)
V1[1]	O8	V3	92.86(6)
V2	O8	V3	169.05(8)
V1	O8	V4	170.77(8)
V1[1]	O8	V4	87.54(5)
V2	O8	V4	86.59(6)
V3	O8	V4	85.72(5)
V1	O8	V5	87.81(5)
V1[1]	O8	V5	170.29(8)
V2	O8	V5	86.93(5)
V3	O8	V5	84.40(5)
V4	O8	V5	82.97(5)
V1	O9	V4[1]	110.92(8)
V1	O10	V5	108.69(8)
V3	O11	V4	115.41(9)
V2	O12	V5	113.40(8)
V3	O13	V5	115.53(9)
V4	O14	V5	113.33(8)
V2	O17	V4	113.22(9)
C23	N22	C21	125.1(2)
C30	N29	C28	126.5(2)
C37	N36	C35	125.8(2)
N31	C30	N32	122.1(3)
N31	C30	N29	117.6(2)
N32	C30	N29	120.2(3)
N25	C23	N24	120.6(3)
N25	C23	N22	117.9(2)
N24	C23	N22	121.6(2)
O27	C28	N33	123.9(3)
O27	C28	N29	122.7(3)
N33	C28	N29	113.3(2)
N38	C37	N39	120.8(3)
N38	C37	N36	122.1(3)
N39	C37	N36	117.1(2)
O34	C35	N40	124.1(3)
O34	C35	N36	121.3(3)
N40	C35	N36	114.6(2)
O20	C21	N26	124.8(3)
O20	C21	N22	120.9(3)
N26	C21	N22	114.3(2)

0 [1] 1-x,1-y,1-z
